# Clinical pattern of nephrotic syndrome and treatment response in children

**DOI:** 10.6026/973206300212257

**Published:** 2025-07-31

**Authors:** Rahul Gedam, Gaurav Kumar Prajapati, Ankit Kumar Pawar, Kamal Malani, Dileep Dandotiya

**Affiliations:** 1 Department of Pediatrics, Netaji Subhash Chandra Bose Medical College, Jabalpur, Madhya Pradesh, India; 2 Department of Pediatrics, Government Medical College Seoni, Madhya Pradesh, India; 3 Consultant Pediatrician, SRPR Multispecialty Hospital, Surajpur, Chhattisgarh, India; 4 PGMO (Pediatrics), Civil Hospital, Bairagarh, Bhopal, Madhya Pradesh, India; 5 Department of Community Medicine, CIMS, Chhindwara, Madhya Pradesh, India

**Keywords:** Nephrotic syndrome, children, steroid response, relapse, renal function, complications

## Abstract

The pattern of presentation, laboratory features, complications and initial therapeutic response in cases of nephrotic syndrome in 1 to 10-year-old children is of interest. A prospective observational investigation was performed on 164 participants for eighteen months. It was carried out in the Pediatric Ward and Outpatient Department from the Department of Pediatrics, Netaji Subhash Chandra Bose Medical College and Hospital, Jabalpur, Madhya Pradesh. It was found that nephrotic syndrome was most common in males, with edema being a universal symptom and a large proportion presented with relapse. Most children responded to the use of steroids, although minorities were steroid-resistant or dependent.

## Background:

Nephrotic Syndrome (NS) has been recognized as one of the main chronic kidney conditions among children, with a rate of 2.92 (1.15 to 16.9) incidence per 100,000 children annually [1]. NS is defined by a group of clinical presentations - *i.e.*, proteinuria, hypoalbuminemia, hyperlipidemia and edema and can lead to life-threatening acute complications, including infections, thromboembolic incidents and acute renal impairment [2, 3]. Clinical presentation and profile of childhood NS differ widely between the developed and the developing world. These differences are largely regulated by environmental factors, infectious disease load and ethnicity, which, in turn, affect the histopathological patterns of the affected patients [4, 5]. Corticosteroids remain the cornerstone in treating NS in children. Steroid-resistant children most often have dismal outcomes despite exposure to potent immunosuppressive agents [6]. Some children achieve long-term remission on a standard regimen of prednisolone; others require agents that reduce the need for steroids, including levamisole, cyclophosphamide, mycophenolate mofetil, calcineurin inhibitors and rituximab and responses are highly variable depending on the case presented [7]. NS is about fifteen times more prevalent among children compared to adults [8] and is thus a prevalent cause of pediatric hospitalization, particularly among younger demographics in countries such as India [9]. About ninety percent of children diagnosed with nephrotic syndrome (NS) also present with idiopathic nephrotic syndrome (INS) and among these, MCNS are the majority (85%) [10]. Relapse rates are highly variable; some patients have many relapses in a year. While the initial Global Research on Renal Disorders in Pediatric Populations had described a recurrence rate of 60%, subsequently reports give rates of up to 76-90%, with up to 50% being frequent relapsers [11]. Infection, particularly of the upper respiratory tract infection (URTIs), has been reported to be an established cause of relapses of MCNS. Their prevention and management can lower the rates of relapses as well as proteinuria, with potential corticosteroid savings [12]. Even asymptomatic UTIs are underdiagnosed and potent causes of disease relapse [13]. Early diagnosis and the histopathological features of this disease have an important place in its prognosis. Knowing the demographic, clinical and pathological features of this disease is helpful in monitoring its progress and for its prognosis [14]. Therefore, it is of interest to examine the pattern of presentation, laboratory features, complications and initial therapeutic response in cases of nephrotic syndrome in 1 to 10-year-oldchildren.

## Methodology:

This study was designed as a prospective observational investigation for eighteen months from March 1, 2021, to August 31, 2022. It was carried out in the Pediatric Ward and Outpatient Department from the Department of Pediatrics, Netaji Subhash Chandra Bose Medical College and Hospital, Jabalpur, Madhya Pradesh.164 Participants in the study included children aged between 1 to 10 years diagnosed with nephrotic syndrome. The calculation of the sample size was performed by using the ratio of occurrence (p = 70.1%) and relative error of 10% at a confidence level of 95% by using the standard formula for sample size calculation in a study on prevalence. Children aged between 1-10 years with clinical presentation of the nephrotic condition, both new and relapse, were part of the research. Children under the age of less than 1 year or older than 10 years, attendants or guardians who could not provide consent and children with co-morbidities other than complications of nephrotic syndrome are excluded. The ethical clearance was obtained from the Committee on Institutional Ethics before initiating the research. Purposive sampling technique was used to enroll 164 consecutive cases of Nephrotic syndrome in children aged 1 to 10 presenting to the pediatric department during the study period. Information was gathered on a predesigned program that recorded demographic details such as age, sex and locality and clinical parameters such as age at onset, presenting complaint, past history, dietary habits and family history. Comprehensive clinical assessment, including vital signs, anthropometry and a systemic analysis, was conducted in every subject. All enrolled children underwent a series of investigations to assess the severity and nature of nephrotic syndrome. Microscopy in routine urine analysis was used to test for proteinuria, red blood cells, pus cells and casts. Urine culture and sensitivity were performed as clinically indicated. Blood workup consisted of triglycerides, cholesterol, lipid profile, albumin and globulin in the serum, serum creatinine and blood urea, electrolytes (sodium and potassium) was done. Imaging was carried out by chest X-ray and ultrasonography of the abdomen. Ultrasonography of the kidneys was carried out in all patients on a routine basis and ascitic fluid examination was performed as and when required.

## Treatment:

Management involved specific and supportive treatment modalities. Nephrotic syndrome's first manifestation was treated with oral Prednisolone 2 mg/kg/day (up to 60 mg) either all at once or in dose for six 1.5 mg/kg for weeks after (maximum 40 mg) on alternate days as a single dose in the morning for another six weeks before withdrawal. On relapse, prednisolone was administered at a daily dose of 2 mg/kg till proteinuria was negative for three days in a row, then 1.5 mg/kg every other day for four weeks. Daily monitoring included urine protein by heat coagulation test, weight, measurement of blood pressure with cuffs suitable for age and measurement of fluid input-output was recorded. Patients who, following four weeks of daily treatment, did not experience remission with prednisolone were defined as having initial steroid-resistant nephrotic syndrome and excluded from subsequent follow-up for this study.

## Follow-up and monitoring:

All patients were followed up daily during hospitalization until discharge. Post-discharge follow-up was performed monthly for six months in kids who had steroid-sensitive nephrotic disease. Follow-up assessments included monitoring for treatment-related complications, clinical response recording and short-term outcome assessment. Home urine protein testing using the heat coagulation method was also taught to caregivers and they were requested to maintain a daily chart of proteinuria, reporting to the hospital in case of positive results for three consecutive days.

## Statistical analysis:

Data were analyzed using SPSS version 20 and MedCalc version 19.5. Quantitative data were tabulated using descriptive statistics, qualitative information, as well as mean ± standard deviation percentages. Univariate and bivariate analyses were used for the purpose of finding associations. Statistical tests like the chi-square test, Fisher's exact test for categorical variables and the Student's t-test for continuous variables were used wherever relevant. A p-value below 0.05 indicated the findings to be statistically significant. For the purpose of finding the strength of association between the variables, Cramer's V and the Contingency Coefficient were also used.

## Results:

The research compared the clinical patterns and treatment outcomes of nephrotic syndrome among 164 children aged 1-10 years. The cohort was divided into equal halves between the ages of 1-5 and 5-10 years, (50.6% and 49.4%), with males 64.6% & females 35.4% (1.8:1 male-to-female ratio). Every subject had edema, whereas secondary manifestations were cough (18.3%), fever (17.7%) and abdominal pain (7.9%). Hypertension existed concomitantly with edema in 17.1% of presentations (Table 1 - see PDF, Figure 1 - see PDF).

## Laboratory and imaging findings:

Hypoalbuminemia was rare (2.4%). Renal dysfunction was suggested by 17.1% abnormal serum urea and 22.0% abnormal serum creatinine levels. Mild-to-moderate ascites were detected on ultrasound in 94.5%, but only 5.5% had gross ascites. There was a strong association between edema with hypertension and mild ascites (71.4%, p=0.027). There existed a robust association between abnormal renal function tests (RFTs) and hypertension (p<0.0001) (Table 2 - see PDF).

## Treatment response and outcomes:

A majority of the children (85.4%) initially responded to steroids, while 12.2% were steroid-resistant. Upon follow-up, 47.1% entered remission, although 25.7% experienced rare relapses. Complications such as urinary tract infection (14.6%) and pneumonia (9.1%) were reported (Table 3 - see PDF).

## Discussion:

The current study assessed the clinical profile, lab results and results of treatment among 164 kids who had nephrotic syndrome. The age range in the present investigation was a close representation of kids aged 1-5 years (50.6%) and 5-10 years (49.4%), being 5.19 ± 2.21 years old on average. This is by the observation of Agrawal and Singh, who gave 6.76 ± 2.8 years old on average, with the maximal age of presentation being 5 and 7 years [15]. Sahana *et al.* also noted a higher mean age of 7.4 years, with 65% of their population in the 6-12 years age group and 31% in the 1-5 years age group [16], which tallies with the observations in the current study. The lower reported mean age (4.08 years) could be due to only steroid-sensitive nephrotic syndrome cases being considered [17]. Pandya *et al.* noted almost equal gender distribution, which can be attributed to the low sample size in their study [18]. The other symptoms, like fever (17.7%), abdominal pain (17.7%) and cough (18.3%), were commonly seen. The development of fever in nephrotic syndrome can be indicative of accompanying illnesses like urinary tract infections and upper respiratory tract infections, or pneumonia, as they are frequent due to the immuno-compromised nature of nephrotic syndrome. Such infections can also act as precipitating factors for relapse. This discrepancy could be a result of varying patient age and the inclusion of relapse cases. There was a strong association between hypertension and advanced age in the current study, indicating that steroid-resistant or atypical nephrotic syndrome could be more common in older children, which is in line with the findings of the International Study of Children's Kidney Disease [19, 20]. About laboratory examinations, hyperlipidemia was uniformly detected, with mean serum cholesterol of 302.94 ± 78.55 mg/dL. There was a statistically significant correlation between raised cholesterol and hypertension, lending support to the hypothesis that hypercholesterolemia is a contributing element to the development of high blood pressure in nephrotic syndrome [21]. Hypoalbuminemia was uncommon in the cohort (2.4%), perhaps due to early diagnosis or less severe proteinuria in most. Serum creatinine was abnormal in 22% and urea in 17.1% of children, suggesting some renal impairment. In the current investigation, the average serum creatinine was 1.29 ± 1.12 mg/dL, greater than usually seen in minimal change disease, raising the possibility of other histologic subtypes [22, 23]. Sharma *et al.* also reported that in nephrotic syndrome, acute kidney damage (AKI) is not uncommon and is more frequent in the steroid-resistant form [24]. Ultrasound scan demonstrated mild-to-moderate ascites in the bulk (94.5%), whereas gross ascites was detected only in 5.5%. There was a strong correlation between the presence of ascites being mild with hypertension and 71.4% of children had both conditions (p=0.027). Ascitic fluid may be easily detected using ultrasound, even with small amounts [25, 26] and in nephrotic syndrome; its presence is well established [27]. Therapeutically, steroid responsiveness was excellent, with 85.4% of the children responding to initial therapy. Steroid resistance was noted in 12.2% and a small number (2.4%) were steroid-dependent.

## Conclusion:

Nephrotic syndrome was most common in males, with edema being a universal symptom and a large proportion presented with relapse. Most children responded to the use of steroids, although minorities were steroid-resistant or dependent. Complications like peritonitis, pneumonia and urinary tract infection were reported and renal impairment in a few instances.
